# ‘I’m trying to tell you this man is dangerous… and no one’s listening’: family violence, parent–school engagement and school complicity

**DOI:** 10.1007/s13384-020-00415-7

**Published:** 2020-11-27

**Authors:** Sue Saltmarsh, Eseta Tualaulelei, Kay Ayre

**Affiliations:** 1 Department of Early Childhood Education, The Education University of Hong Kong, Tai Po, New Territories, Hong Kong SAR; 2 Early Childhood Curriculum and Pedagogy, School of Education, University of Southern Queensland, Springfield Campus, Springfield Central, QLD Australia; 3 Early Childhood Studies, School of Education, Edith Cowan University, Perth, WA Australia

**Keywords:** Parent engagement, Relationship dissolution, Divorce, Family violence, Gender, Complicity

## Abstract

This paper presents a case study of one mother’s experience of engaging with her children’s schools after leaving a long-term relationship characterised by years of family violence perpetrated by the children’s father. We interviewed Bernadette as part of an ongoing study of parents’ experiences of school engagement during family separation and divorce. Her family circumstances and the role the children’s schools played in that story merit consideration by educators, school leaders and education policy makers. Informed by theories of everyday cultural practices and sociological studies of gendered power relations in education, we argue that gender politics and organisational strategies for keeping parents ‘in their place’ can significantly contribute to systemic failures and school cultures that reinscribe the effects of family violence.

## Prelude: conversations Australian educators need to have

In a telephone interview that took place in late 2019, our research team spoke with Bernadette^1^ about her experiences of engaging with her children’s schools after ending her relationship with the children’s violent father. Her experiences with school staff during this period of family upheaval, described in greater detail in a later section of this paper, were unsatisfactory in numerous critical and troubling respects, raising questions about the ways that schools are uniquely positioned in the lives of families, including families in crisis. Bernadette’s experience underscores an urgent need for a national conversation about school policies, procedures and everyday practices when dealing with children and parents living with or fleeing family violence.^2^ While research concerning schools and family violence tends to focus on identifying signs of child neglect or other forms of abuse, understanding professional reporting obligations, and cooperation between social services, less has been written in the education research literature about schools as sites of intervention and support, or as sites where staff, students, routines, policies and procedures may be directly impacted upon by family violence (Eriksson et al. 2013a, 2013b). When parental relationship dissolution and negotiation of parenting responsibilities are the subject of legal proceedings, some researchers have observed that ‘studies on the intersection between pre-school/school and family law disputes seem virtually non-existent’ (Eriksson et al. 2013b, p. 2). Where there is a history of family violence, such disputes carry additional risks of physical, emotional and psychosocial harm, and school may be a critical site for ensuring children’s safety at what is a fraught and potentially dangerous time (Jaffe et al. 2017).

We situate this paper, then, within the broader context of family violence and dangers faced by women and children living in and fleeing violent homes, and informed by feminist scholarship from disciplines including sociology of education, family law and family violence studies over the past two decades (see, for example, Fine 2012; Kenway and Fahey 2008; Kenway and Fitzclarence 2005; Lapierre 2008, 2010; Lapierre et al. 2018; Luttrell 1997; Skinner et al. 2013; Weiss et al. 1998). These feminist scholars have highlighted how, for example, the unequal power relations between men and women together with gendered social expectations, disproportionately place responsibility for children’s safety and wellbeing onto mothers. Simultaneously, such expectations rely on deficit discourses in relation to mothers who are subjected to violence in their intimate partner relationships (Lapierre 2008, 2010; Lapierre et al. 2018). While we acknowledge that not all family violence is perpetrated by men, research is clear that ‘Most of the high risk cases with a history of serious violence will involve fathers as perpetrators of violence’ (Eriksson et al. 2013a, p. 87).

As this manuscript was nearing completion, news broke of a family homicide in Brisbane that sparked intense public debate about the seemingly intractable problem of family violence in Australia. Details unfolded in the days following 19 February 2020, when 31-year-old Hannah Clarke and her three young children were in their car on the way to school when the children’s father, recently separated from Hannah, forced his way into the car, doused Hannah and the children with petrol and set them alight, before killing himself with a knife. The horrific deaths of Hannah and her children prompted a national outcry from support and advocacy groups, survivors, domestic violence experts, community and political leaders (Chang 2020).

Many argued that violence against women in Australia has reached epidemic proportions, a contention supported by national statistics showing that between 2014 and 2016, one woman was killed in Australia every nine days by a current or former partner (Australian Institute of Health and Welfare 2019). Others observed that such tragedies typically come about in the context of identifiable histories of abuse and predictable patterns of behaviour. As one study noted, ‘domestic homicides are characterised by predictable aetiologies and behaviour…and can therefore be considered preventable deaths’ (Butler et al. 2017, p. 129). This view is echoed by the Australian Domestic and Family Violence Death Review Network finding that ‘…domestic and family violence deaths can be seen as largely preventable’ (2018, p. 31). In the midst of these debates, however, a senior officer was stood aside from the investigation by Queensland Police following comments that were widely criticised as victim blaming, and symptomatic of entrenched societal attitudes that continue to hold women, rather than men, responsible for the violence perpetrated against them (McGowan and Smee 2020; Woolley and McElroy 2020; Truu 2020).

Shortly after the deaths of Hannah Clarke and her children, Bernadette contacted our research team again, saying that the recent media accounts had caused her to reflect on how her and Hannah’s stories of family violence were ‘almost exactly the same, word for bloody word’. From years of enduring coercive control and violent outbursts, social isolation, stalking and even abduction of one of the children post-separation, to having her fears for her own and her children’s safety not being taken seriously by authorities at critical points, the two women’s stories share numerous similarities. ‘The only real difference’, Bernadette remarked, ‘is that one of us is lucky enough to still be alive’.

In Bernadette’s case, a significant complicating factor in her efforts to keep her children safe during the post-separation period relates to interactions with school personnel. We take seriously the gravity of these experiences, and aim here to explore how everyday gendered school-based practices and interactions between parents, children and school personnel are implicated in outcomes upon which people’s lives may depend. We offer an analysis of Bernadette’s story with a view to better understanding the interface between families and schools during times of crisis associated with family violence, relationship dissolution and parenting disputes. In so doing, we call for a collective rethinking of school responses to families in these circumstances, in order to ensure that they are part of solutions rather than complicit in putting children’s and families’ wellbeing and lives at risk.

## Schooling and family violence in context

Experiences of parents leaving violent relationships are understood here within the broader context of parental relationship dissolution (also discussed in terms of separation and divorce), the potential impacts of family violence on children’s schooling and how schools engage with affected children and parents. We incorporate parental separation and divorce into our discussion of family violence for three main reasons. Firstly, the main focus of the study from which this paper is drawn concerns parent–school engagement during separation and divorce. It is within this context that participants, including Bernadette, volunteered to be interviewed. In Australia, 47.3% of all divorces in 2018 involved children under the age of 18, affecting 42,523 children in that year alone (Australian Bureau of Statistics [ABS] 2019). The most recent figures from the Organisation for Economic Cooperation and Development (OECD) report even higher global percentages, with children under the ages of 18 affected on average in 56.2% of all divorces worldwide (OECD 2015).

Secondly, while not all parental separation and divorce involves family violence, the extent of intimate partner violence, together with the issue of under-reporting, is widely acknowledged (Meyer and Frost 2019; Morgan and Chadwick 2009). Globally, intimate partner violence affects between one in three and one in four women from the age of 15 years (Meyer and Frost 2019). In Australia, the most recent Personal Safety Survey (PSS) conducted by the ABS in 2016 found that some 2.2 million adults had experienced intimate partner violence since the age of 15 (ABS 2017), and of those women whose most recent incident of physical assault had been perpetrated by a male partner, only 34% had been reported to police (ABS 2020). More concerning still are the numbers of children worldwide affected by family violence, which in some fields is commonly referred to as ‘the most pervasive, yet least recognised, human rights abuse in the world’ (Brickell and Garrett 2015, p. 929). While children’s experiences of family violence are not consistently captured in any comprehensive Australian data, the most recent PSS indicates that ‘around 418,000 women and 92,200 men who had experienced violence from a previous partner said the children in their care had witnessed this violence’ (AIHW 2019, p. 4). Other research shows that approximately 6% of children are exposed to family violence annually, with 16–18% of children affected during the course of childhood (Carson et al. 2020; Hamby et al. 2011).

Thirdly, the relationship status of current and former intimate partners with children is highly relevant to schools with respect to issues of safety and vulnerability. Studies in the US (see, for example, Beck and Raghavan 2010; Greenberg et al. 2006; Jaffe et al. 2017; Kelly and Johnson 2008) suggest that among separating or divorcing couples involved in court-related mediation and custody proceedings, the incidence of family violence is disproportionately high, affecting up to 80 percent of these families. School awareness is critical, particularly given that parents who inform schools about changes in relationship status, living and parenting arrangements, mediation or custody proceedings may be directly affected by family violence, without necessarily disclosing these experiences to schools (Lloyd 2018; Stanley et al. 2012). Importantly too, family violence research is clear that the period preceding and shortly following relationship dissolution between intimate partners is a particularly risky time for family violence (DeKeseredy et al. 2017; Morgan and Chadwick 2009), including risks of child abduction (La Kam 2016) and paternal familicides (Cullen and Fritzon 2019; Declercq et al. 2016; O’Hagan 2014). According to one recent Australian study ‘Actual, imminent or threatened marital separation was a factor in about half…of all familicide cases, while around one quarter…occurred against a backdrop of contested custody proceedings’ (Cullen and Fritzon 2019, p. 976).

The numbers of children affected by parental separation, divorce, and family violence, and the seriousness of associated implications and risks, suggest that educators and school personnel need to be informed about and sensitive to the relevant issues (Colpin et al. 2004). Researchers concerned specifically with the role of schools in the lives of children and parents affected by family violence point out that ‘Given the multiple effects of domestic violence, teachers and support staff in schools need to be equipped with knowledge, understanding and skills to identify and respond to internalized and externalized symptoms’ (Lloyd 2018, n.p.). While a detailed discussion of the effects of family violence on children is beyond the scope of this paper, it is the subject of a significant body of research (see, for example, Callaghan et al. 2018; Katz 2016; Katz et al. 2020; Lloyd 2018; Schneider et al. 2020), including research indicating that schools can be an important site of safety, continuity and support for children (Ellis et al. 2015; Lloyd 2018; Stanley 2011). As one research team puts it, ‘For children who experience problems in their family, pre-school and school can offer relief in difficult life situations’ (Eriksson et al. 2013b, p. 1). However, this is not necessarily the case, with researchers also highlighting that difficulties experienced by children at home can be exacerbated at school, particularly where family violence is implicated in children having difficulties with learning, behaviour, attendance and participation (Eriksson et al. 2013a; Lloyd 2018).

The impact of relationship dissolution and family violence on parent–school relationships is also situated in the context of parent–school engagement discourse, which is typically constructed by policy makers, educators and parent advocacy groups as the primary means of ensuring children’s educational success (Saltmarsh 2015; Saltmarsh and McPherson 2019). However, some critics suggest that these discursive and policy rhetorics assume an idealised parent and family, contributing to common-sense notions that shape the everyday practices of schools towards parents (see, for example, Goodall 2019; Kainz and Aikens 2007; McKay and Garrett 2013; Saltmarsh 2015; Saltmarsh and McPherson 2019). Research shows, for example, that parents and schools may struggle to maintain positive relationships when difficult or contentious issues arise or remain unresolved (MacFarlane 2008, 2009; Barr and Saltmarsh 2014, Saltmarsh and McPherson 2019). That some parents may fare less well than others in these interactions can also be attributed to disparities associated with gender, race, culture, socioeconomic background and other identity categories.

According to some, the explanation for such disparities lies in the ways that common sense has been generated about what it means to be a ‘good’ or ‘responsible’ parent who provides a ‘good’ home for their children (Goodall 2019; Kainz and Aikens 2007; McKay and Garrett 2013; Saltmarsh and McPherson 2019; Vincent et al. 2010). These common-sense understandings function ‘as a dominant discourse that shapes individual identities, identities that are then publicly prescribed and that reify stereotypes of expected behavior’ (Kainz and Aikens 2007, p. 301). As feminist scholars have argued, this can be especially true of gender stereotypes and dominant discourses of mothers and motherhood (Gillies 2006; Peters 2012), and is further compounded by social class bias that constructs working-class parents as morally deficient, and ‘underpins disrespectful and sometime hateful depictions of working class mothers as lazy, ugly, stupid and dangerous’ (Gillies 2006, p. 283). This feeds into what has been described more broadly as the ‘culture of mother-blame’ (Peters 2012, p. 121), in which mothers are predominantly held responsible for children’s development, behaviour, educational success and wellbeing. As Goodall observes, placing these expectations for securing children’s outcomes onto mothers takes place alongside beliefs about their ‘inability to carry out these functions’ (2019, p. 8). Given that the vast majority of victims and survivors of family violence are women (AIHW 2019), the ways in which schools interact with mothers—including mothers whose domestic circumstances fall outside idealised, normative expectations—has particular salience.

Feminist research in education has shown, for example, that even in ordinary circumstances it is predominantly mothers who shoulder most of the responsibility for children’s schooling. Whether it be choice of school (Aitchison 2006, 2010; Leyton and Rojas 2017), assisting with homework (Hutchison 2012; O’Brien 2008; Shuffleton 2017), maintaining parent–teacher relations (Blackmore and Hutchison 2010) or negotiating solutions to problems with teachers or peers (Vincent and Martin 2002; Vincent and Ball 2007; Vincent 2017), it is typically mothers whose physical and emotional labour, sometimes discussed in terms of social and emotional capital, is invested in supporting children’s learning and schooling experience. As Maeve O’Brien (2008) summarises, this can involve managing multiple and at times competing demands, including:

…visiting and contacting schools, attending meetings, organising children for the school-day, organising and providing transport, supporting children through their assessment tests, helping with homework, listening to children talking, listening out when children did not talk, finding opportunities for communication, and in general making sure that children were well cared for physically… (p. 141).

For mothers parenting during times of personal or familial crisis, such responsibilities—especially when undertaken alone and with no or limited support—can be additionally burdensome. As Kainz and Aikens (2007) point out in their genealogy of parent involvement discourse, ‘this discourse and its related policy and practice reflect expectations for family involvement and family structure (i.e., two-parent families) that ignore and ultimately harm those with divergent cultural perspectives and family structures’ (p. 302).

In light of tensions that occur at the convergence of normative gender discourse, idealised expectations of mothers, and parent–school engagement, it is not difficult to imagine that mothers trying to navigate the complex circumstances of family upheaval—and more so in the case of mothers who are fearful for their own safety and that of their children—might encounter specific challenges. They may struggle to convince schools of the effects of family circumstances on children’s attitudes, behaviour and attendance, and may find that schools are unclear about their roles, entitlements and obligations pertaining to family law issues (Cooper et al. 2012; Eriksson et al. 2013a, 2013b; Peters 2012). They may experience difficulty in being heard and taken seriously on important matters, or have difficulty accessing teachers or school leaders (Barr and Saltmarsh 2014; Saltmarsh 2015). Conversely, they may find that persistent requests for counselling, information or resolution of specific problems results in being ‘temporarily placated, dismissed or deemed deficient as parents’ (Saltmarsh and McPherson 2019).

Engaging with families during such times can also present challenges for schools, particularly where custody, guardianship or Protection Orders are before the courts (Cooper et al. 2012; Eriksson et al. 2013a, 2013b). Schools can be impacted in multiple ways when parenting disputes or concerns about family violence spill over into the role of schools (Cooper et al. 2012; Eriksson et al. 2013a, 2013b), and this problem can be exacerbated by the ways that courts and other agencies sometimes treat schools as de facto neutral or informal sites for mediation of conflict (Eriksson et al. 2013a, 2013b). This can take the form of expectations that school staff will play a role in sharing information between parents, or stipulating school as the place where handover arrangements of children are expected to occur. In cases involving ongoing family violence investigations, there may also be expectations by family law social workers, for example, that schools will facilitate interviews with children and teachers (Eriksson et al. 2013a, 2013b).

Another crucial issue for schools highlighted by family violence research is well-documented patterns among men with a history of intimate partner violence, coercive control and stalking behaviour, who may use schools and other institutions as a means of continuing to victimise former partners (Cattaneo et al. 2011; Miller and Smolter 2011; Schandorph et al. 2019). ‘Paper abuse’ and ‘procedural stalking’—terms largely unfamiliar in the education literature—refer to abusive tactics of using courts, child protection, education and other bureaucratic systems to punish and control former partners (Miller and Smolter 2011). For example, ‘Some abusers make false reports of child abuse against mothers, claim that the mother is unfit as a parent, or make false complaints of interference with or denial of visitation’ (DeKeseredy et al. 2017, p. 118). Such claims may be made through court and child protection systems, costing mothers time and money to defend in court, undermining their credibility, undermining maternal–child relationships, and in some cases, resulting in reduction or loss of care time. Schools can be unknowingly conscripted into enabling this conduct. Abusers may attempt to manipulate school staff in the interest of, for example, influencing favourable expert testimony by teachers and counsellors, creating impressions of the other parent as uncooperative, abusive or unfit, or refusing to cooperate with permission for school-related activities such as extra-curricular activities (see DeKeseredy et al. 2017; Miller and Smolter 2011; Saunders and Oglesby 2016; Schandorph Løkkegaard et al. 2019).

In the sections that follow, we consider how these issues are played out in one mother’s account of her attempts to engage with her children’s schools during a period of post-separation from a violent, coercive controlling partner. In particular, we are interested in how discourses of gender and parenting converge at the site of the school, erecting seemingly impenetrable barriers to Bernadette’s endeavours to secure her children’s safety and wellbeing during a time of family crisis and profound risk. Our analysis is informed by poststructural and cultural theories concerned with power relations, and the ways in which relationships of power act not only on individuals, but also upon the actions of others (Foucault 1982). For Foucault, educational institutions are sites in which power is operationalised through a range of institutional procedures, hierarchies and regulatory practices that construct, categorise and discipline individual subjects. Michel de Certeau (1984) brings these ideas into play with respect to the interplay between ‘the institutional strategies that structure, conceal and maintain the operations of power [that] are used to keep those without a ‘proper place’ within the institution at a distance’ (Saltmarsh 2015, p. 41) and the tactics through which individuals exercise agency and resistance. For Certeau, the interplay of strategies and tactics is implicated in and productive of cultures that are permeable and dynamic.

We find these insights useful for exploring how gendered power relations (Arnot 2002; Goodall 2019; Osgood and Robinson 2019; Youdell 2019) are embedded within institutional strategies and school cultures that shape interactions between parents and school personnel. We understand these interactions between schools and parents such as Bernadette as indicative of ‘how gender intersects with social class, race, sexuality and so on to position women and girls within cultural systems of difference that cast them as subordinate, inferior or in some sense other’ (Osgood and Robinson 2019, p. 3). Thus we argue that gender politics and institutional strategies for keeping parents—and in particular, mothers—‘in their place’ can significantly contribute to systemic failures that reinscribe the effects of family violence, with damaging consequences for children and families.

## Methodological and ethical considerations: sensitivities, risks and responsibilities

This study arises from a larger program of research concerned with parent–school relationships, including perspectives of parents, educators and school leaders. Findings analysed here are drawn from a suite of telephone interviews conducted in an ongoing Australian study of parent–school engagement during separation or divorce. Following approval from the university Human Research Ethics Committee,^3^ participants were recruited through national parent representative organisations,^4^ who circulated links to information about the study through their normal channels of communication with parents such as newsletters, websites, Facebook and Twitter. Some word-of-mouth recruitment also took place, as participating parents occasionally referred others to the research team. Prospective participants used a secure link to access additional project information, leaving a contact number, first name or pseudonym (if they preferred to participate anonymously), and their preference of dates and times for the interview. All interviews were conducted by telephone to provide additional privacy and to maximise convenience to participants.

Several ethical considerations pertaining to this project merit discussion. The first pertains to the sensitive nature of the research topic, bearing in mind that for many people the dissolution of a relationship is often complex and emotionally distressing, and more so when children are involved. That being the case, inclusion criteria specified that participating parents had been separated from their former partners for at least 12 months prior to interview. While we acknowledge the potential for participants to still experience varying degrees of distress when talking about such events even after 12 months, we were also mindful that it was important to capture parents’ perspectives while their interactions with schools during the post-separation period were able to be recalled readily. With these sensitivities in mind, participants were reminded prior to commencement of interviews of the voluntary nature of their participation, and of their entitlement to take breaks during the interview, to decline to answer any questions they preferred not to answer, to end the interview and/or withdraw from the study at any time. Participants were provided with multiple contacts for support services that could be accessed should they experience distress during or after participation, and were also encouraged to seek advice from their own medical practitioners.

Another ethical consideration in studies of this sort is confidentiality. While the research team employed usual practices in education research such as anonymising transcripts and allocating pseudonyms for individuals and schools, additional measures were taken to protect participants’ identities. For example, we used a coding system for the allocation of pseudonyms, and confirmed with participants that they were neither currently nor previously known by the pseudonym allocated to them. Pseudonyms were used for any names of individuals or schools, and a decision was made not to name specific towns or states/territories within which participants, their families, children or former partners/spouses live. Instead, where it has been necessary to discuss aspects of a school community, we have used terms such as ‘capital city’, ‘regional city’ or ‘rural community’ rather than giving further specifics. We also elected not to include information such as children’s ages or sector of schooling in which they are enrolled. Prior to commencement of interviews, we discussed privacy and confidentiality issues with each participant, giving them the opportunity to ask questions and to flag any concerns they might have in relation to the publication of details pertaining to their personal and family circumstances. In Bernadette’s case, because there are ongoing sensitivities pertaining to her own and her family’s safety, she was given the opportunity to review both her interview transcript and the article manuscript prior to submission.

While unique in some specific details, Bernadette’s experience of family violence is not particularly unusual. A recent report by the Australian Bureau of Statistics (ABS) shows that nearly a quarter of women in Australia (23%, 2.2 million) have experienced emotional abuse by an intimate partner, and one in six (17%, 1.6 million) have experienced sexual and/or physical violence by an intimate partner (ABS 2016). Concerning too is that women in Bernadette’s age bracket are over three times more likely to experience sexual and/or physical violence from an intimate partner than women age 35 or over (Australian Institute of Health and Welfare (AIHW 2019). We are also mindful that while our research team has expertise in researching sensitive issues including violence in schools, childhood trauma, and various forms of marginalisation and social disadvantage, we are neither therapists nor counsellors. Hence, we see our role in documenting the experiences of parents during difficult periods of family life and school engagement not as providing help, advice or support to individual participants, but rather as a means of sharing their experiences in ways that may open up professional and policy dialogues leading to meaningful change. As Jane Kenway and Johanna Fahey recount in their study with marginalised young women and their mothers, many of whom had experienced sexual abuse and family violence:

We were disturbed and moved by these stories and humbled by the fact that we were trusted with them. Unable to provide any of the type of help that might really help in the immediate circumstances, we were determined to ensure that these girls’ and women’s gruelling, powerful and poignant stories would be aired in the public fora to which we had access…with a view to them joining the clamour of voices that seek to ensure that the issue of women, girls and violence does not slip off the public policy agenda (Kenway and Fahey 2008, p. 642).

Our research team acknowledges participants’ willingness to share with us their deeply personal accounts of what most had experienced as difficult and at times profoundly painful family circumstances. Although Bernadette’s was not the only participant story that involved custody disputes or family violence, it underscores the need for greater attention among education researchers, practitioners and policy makers to the intersection of family violence and schooling. As such, it merits the close attention made possible by considering it as a case study located within a broader constellation of questions around gender, family violence and the complicity of social institutions such as schools.

## Bernadette’s story: a case study within a bigger picture

Bernadette is in her 30s, and the mother of Katie, who is in the early years of high school, and Narelle and Nina, both of whom are in primary school. Approximately 18 months prior to interview, Bernadette left a long-term relationship with her children’s father, whose behaviour she describes as coercive, controlling, violent and unpredictable. Increasingly fearing for her own safety and that of her children, she left the relationship, moved to a nearby regional city and enrolled her daughters in new schools. Worried that cutting off contact with the children might result in violent reprisal from their father, she initially agreed to him spending time with them after school one or two afternoons each week. However, when Bernadette learned that he had arranged to meet secretly with the children and the school counsellor, and had been coaching the children to tell the counsellor that ‘mum had been beating them, and mum had been abusing them, and they wanted to live with dad’, Bernadette brought his unsupervised contact to a halt until legally binding parenting orders could be issued by the court. When more serious threats to the family’s lives and physical safety came to light, Bernadette filed an urgent application to the court for what in some Australian states and territories are referred to as Apprehended Domestic Violence Orders or Protection Orders.^5^

The court issued the Protection Order within 24 hours, and Bernadette immediately notified both schools and provided copies of the relevant documentation. She emphasised that police should be called should the children’s father come to the schools or approach the children, both of which were prohibited under the Protection Order. One week later the father entered school grounds at the end of a school day, and after aggressive encounters with a family member and Nina’s teacher, left with all three children. Despite school staff being aware of safety concerns for the children, no calls were made to police, nor was the father prevented from leaving school grounds with the children. Because no custody orders were yet in place, and police had yet to serve the Protection Order, local police advised that there was little they could do. That afternoon, they attended the father’s house, served the Protection Order and secured a verbal agreement by him to return the children to their mother in several days’ time, leaving the children in his care and Bernadette to endeavour to retrieve them on her own three days later.

When Bernadette returned to collect the children as agreed, the father only permitted the younger two to leave, insisting that Katie now wanted to live with him, and refusing to let Bernadette see or speak to Katie. The following day he contacted the high school, reporting that Katie now lived with him full-time. Bernadette lodged an urgent application for interim custody orders, requesting that Katie be returned and all three children remain in her care while their case made its way through the family court system. Two months passed before the matter was heard in court, during which time Katie was not permitted by her father to see Bernadette or her sisters, and was only permitted to speak with them by phone on a few occasions while the father listened in.

During this time, Bernadette contacted Katie’s high school on numerous occasions, in person, by phone and in writing. Her primary concern was Katie’s safety and wellbeing, given that she was now in the care of someone who was the subject of a Protection Order on which Katie and her sisters were named persons. Bernadette asked that Katie be supported by the school counsellor, and that she be permitted to meet with Katie at school. After multiple requests, the school eventually agreed to facilitate the meeting, which fell through when Katie was informed and followed instructions from her father, of whom she was afraid, not to attend the meeting. Bernadette’s requests for support at school for Katie, and for herself as a parent trying to protect her child from a known domestic abuser, were ultimately unsuccessful. Deeply worried about Katie’s safety and wellbeing, Bernadette asked to meet on her own with the school counsellor and the school principal, hoping that she might have the opportunity to discuss the family situation with them. These requests too, including those put in writing, were either treated dismissively or ignored altogether. She recalls being told by school staff on one occasion that she ‘might just have to accept the situation of Katie living with her dad’, and on another being told that the school ‘normally only deals with the parent with whom the child resides’. Despite repeated requests, she was not provided with any further school reports or information about Katie’s academic progress or wellbeing.

Two months later, the family court ordered that Katie be immediately returned to Bernadette’s care, and that only supervised contact between the children and their father be permitted. Upon her return, Bernadette discovered troubling signs that Katie’s mental health had deteriorated significantly during the time she had been with her father. Her school backpack contained several kilograms of rotten food—lunches that Katie had been taking to school but not eating, because ‘she said she was ashamed to eat’. Katie also confided that she had been self-harming while at school, stapling her fingers together in class and cutting her legs on the playground during recess and lunch, and that she had been experiencing suicidal thoughts. Bernadette sought immediate help from her family doctor and mental health professionals, and notified the school, feeling certain they should be aware of these developments.

Katie also disclosed that she had been informed by the school counsellor that notes from their conversations could be used in court to support the claims by Katie’s father that she wanted to live with him. Upon learning this, Bernadette formally withdrew her consent for Katie to meet with that particular counsellor. She requested instead that the school provide some indication of how Katie could be appropriately supported and kept safe while at school. She reiterated her concerns both about the physical danger posed by Katie’s father, and the complex trauma and mental health issues for which Katie was now being treated. However, her encounters with the school continued to be entirely unsatisfactory. On rare occasions she was able to speak with them, but both the year coordinator and the deputy principal she had been directed to contact were both openly dismissive of Bernadette. She recalls feeling that there was a significant degree of sexism in the attitudes she was encountering, and that despite being a university educated professional employed full-time in the public service, and having ‘dealt with them as I would in a professional environment’, she was being shoved aside and treated as an incompetent parent or as a ‘hysterical ranting woman’. The majority of Bernadette’s phone calls and voice messages were not returned, and emails documenting explicit concerns about Katie’s mental health and self-harming at school went unanswered. She concluded that no one at the school cared about her child’s safety and wellbeing, and ultimately decided that her only option was to move to another community and school in order to protect her children.

## ‘I’m trying to tell you, this man is dangerous…and no one’s listening: cultures, complicity and family violence

Bernadette’s story is detailed and complex, and we acknowledge that the issues it raises merit careful analysis that are not possible to address in a single paper. While some themes from Bernadette’s story are analysed in a suite of papers currently in review, we have chosen here to focus on three key issues that highlight procedural and systemic practices that potentially reinscribe the effects of family violence at the site of parent–school engagement. The **first** of these issues pertains to whether school staff are sufficiently knowledgeable about family violence and their responsibilities in relation to at-risk families and children who are its victims/survivors. As already noted, research concerning the impact of family violence on schools is limited (Eriksson et al. 2013a, 2013b), as is jurisdictional and evidence-based guidance for schools regarding their attendant legal and ethical obligations (Cooper et al. 2012). However, researchers argue for the importance of school staff who both understand family violence issues and their effects on children, and who can respond with sensitivity (Colpin et al. 2004; Davies and Berger 2019; Lloyd 2018; Sterne and Poole 2010). Confusion over roles, responsibilities policies and procedures, together with what some have argued is a lack of teacher preparation and professional development around family violence issues (Davies and Berger 2019), all play a part in shaping the outcomes for affected children and families. Bernadette herself noted that by treating family violence as a private family matter, her children’s schools had not been prepared or willing to take decisive action when circumstances necessitated:

I wish the school, both schools were more prepared for these types of situations. I wish that they had more authority to step in. Because I think that’s a real problem, they think they’ve got no authority, they think it’s not their place.

Some researchers explain the kinds of responses Bernadette encountered in terms of school staff developing strategies for avoiding and managing conflict and violence between parents through processes of *distancing* and *disciplining*. Distancing, for example, may involve constructing family violence and conflict ‘as something outside of staff responsibilities’ (Eriksson et al. 2013b, p. 115), or it may involve gendered or racialised othering that normalises violence and conflict by constructing parents, socioeconomic and cultural groups as different, dysfunctional or deviant. Bernadette’s experience highlights how such distancing, combined with a lack of understanding on the part of school staff, can contribute to unhelpful responses when faced with challenging family violence issues. For example, despite formal notification and regular updates about Bernadette’s family circumstances, the primary school attended by her younger two children had no action plan to implement when confronted with her aggressive former partner on school grounds. As Bernadette explains:

…they had no plan in place for responding to that threat, which they knew was a threat because I’d told them a hundred and fifty times, and given them all the paperwork that they needed to see that the threat was real. Yeah, it was just dismissed from day one as ‘Oh she’s just being dramatic’, and then when it happened [when he took the children from the school grounds and refused to return them] it was like, ‘Oh well sorry about that, you’ll have to go to court’.

Here, distancing becomes a mechanism for displacing the school’s responsibility for the children’s safety while on school grounds, onto Bernadette and the court system. The same sexism, inaction and minimisation so often encountered by women when bringing family violence matters to the attention of police or before the courts (DeKeseredy et al. 2017; LaPierre 2008; Monk 2017; Saunders and Oglesby 2016) is reinscribed here by schools that dismiss her safety concerns as ‘dramatic’ rather than taking them seriously and responding accordingly.

Policy and procedure are also salient not just because non-existent or poorly implemented policy and procedure potentially puts children at additional risk, but also because of the ways that policy and procedure can be manipulated by abusers in well-documented patterns typical of post-separation family violence. Indeed, Bernadette’s former partner persistently provided false or misleading information to the children’s schools, and attempted to manipulate meetings with school counsellors as a way of formalising his accusations against Bernadette in the absence of any evidence. Such conduct is consistent with ‘paper abuse’ and ‘procedural stalking’ identified in the family violence literature as a common form of coercive attempts to control partners who have left abusive relationships (Cattaneo et al. 2011; Meyer and Frost 2019; Miller and Smolter 2011; Schandorph Løkkegaard and Elklit 2019). However, when taking place in the context of schools, where multiple, interconnected stakeholders—office staff, counsellors, teachers, principals, liaison officers, etc.—are potentially conscripted into the abuser’s agenda, we suggest the term ‘coercion of organisational networks’. Importantly, schools whose staff are unaware of such patterns of abuse are vulnerable to helping, even if unintentionally, to facilitate and perpetuate them.

While we acknowledge that expertise in family violence is not the primary role or function of schools, understanding its potential impacts on children who experience it is necessary in order to meet their learning and support needs. This raises a **second** key issue, which is that Katie’s school neither recognised nor responded when presented with evidence of the risks posed by her family circumstances and the social, emotional and mental health issues with which she was grappling. Bernadette recounts being told on several occasions during the two months that Katie was being withheld by her father, that she might need to accept that if Katie wanted to live with her father (as he had claimed, and had been coaching her to claim), and that ‘the family court’s probably not gonna side with you on this one’. By minimising a case of parental separation involving family violence as a mundane disagreement between parents about custody matters, the school missed important opportunities to recognise that Katie herself was a victim of her father’s ongoing abuse. The sexist indifference encountered by Bernadette was thus extended to Katie, further reinscribing at school the gendered family violence and coercive control to which Katie was being subjected while being withheld by her father. Bernadette’s response to one such interaction with school staff underscores her frustration:

I’m telling you, this is not just a normal custody issue, not just a normal family break-up. This person is abusive and dangerous, and I have a Protection Order that goes for five years because the court has also recognised that he is dangerous. I don’t understand…And I was like ‘Do you understand psychological abuse? Like, do I need to explain that to you?’

Despite the education-specific research literature concerned with recognising signs of children’s exposure to various forms of abuse, the professional ethics and duty of care towards students, identifying and supporting children experiencing mental health issues (see, for example, Frauenholtz et al. 2017; Sterne and Poole 2010; Webster and Whelen 2019), and the provision in Australia of mandatory reporting requirements (Oates 2018), a number of key staff, including counsellors and school leaders at Katie’s school persistently overlooked the impact of her family circumstances on her health and wellbeing. Bernadette confirmed that no professional social work or care services were involved with Katie’s case management at the school, nor did she receive recommendations or referrals to such services. As already noted, requests for support from the school counsellor were initiated by Bernadette rather than the school, and these ultimately proved unsuccessful (in Bernadette’s case) and counter-productive (in Katie’s case). When Bernadette made formal requests for support and assistance after learning that Katie had been self-harming at school, she was sometimes treated dismissively or sometimes ignored altogether. As Bernadette recalls:

Bernadette: …when Katie was finally ordered to be returned to me by the court, she came back with an eating disorder and had been talking about self-harming. And said she had depression, and said she hated her life, and things like that…and um, and I reported those concerns to the school and no action was taken at all, even though the reports of self-harm were that she was cutting herself at school, on school grounds, in class…and I reported that to the deputy, and there was just nothing. No response, no nothing.

Interviewer: No response to you reporting to the school that your child had been self-harming at school?

Bernadette: No, no response.

Interviewer: No follow up by a school counsellor with the child?

Bernadette: Nope. Nothing. It was just ignored… Nothing. Not a phone call, I tried to call them and left probably five or six voicemails over a period of about a week, I put it in writing. Nothing. I didn’t get anything.

Importantly, organisational strategies of distancing such as these also have a disciplinary function (Foucault 1982), reinforcing discursive gender hierarchies and institutional strategies (Certeau 1984) that relegate both mother and daughter to a category of irrelevance. This corresponds with our work elsewhere (Saltmarsh and McPherson 2019) that shows how assumptions and attitudes can be tightly interwoven in the ways that schools interact with parents, and can be seen as a means by which families—and in particular mothers—are disciplined, governed or policed (Kainz and Aikens 2007; Vincent and Tomlinson 2006; Barr and Saltmarsh 2014; Saltmarsh 2015; Saltmarsh and McPherson 2019). Such disciplining of parents ‘at times takes place through institutional strategies such as unidirectional communication or lack of consultation, and at other times through dismissive, obstructive or exclusionary practices that keep parents ‘in their place’…in relation to the school’ (Saltmarsh and McPherson 2019, p. 12). It also holds in place ‘a code of silence surrounding domestic violence’ (Weis et al. 1998, p. 61)—a silence into which many women and children are socialised, but which is further ‘maintained and hardened by most of the institutions which structure the lives of these females, including family, school, community and the justice system’ (Weis et al. 1998, p. 67). The school’s refusal to respond to Bernadette’s pleas for support for Katie does more than distance and discipline the parent. In failing to take seriously Katie’s vulnerability and the risks to her physical and mental health, the school’s silence reinscribes, reproduces and amplifies the effects of the gendered family violence that she has already experienced. This institutional silence carries a powerful message to both mother and daughter about their place in the discursive hierarchy, and ‘effects that most damaging of all violences—erasure’ (Saltmarsh 2012, p. 29).

This raises a **third** key issue of concern, which is the gendered nature of school and professional cultures, and their complicity in the production of violence and its effects. As feminist research on school violence and complicity has argued, violence is embedded in the ‘culture and power relationships’ of schools (Kenway and Fitzclarence 2005, p. 47). The reiteration of systemic and everyday gender discrimination faced by women and mothers—well-documented both in the education *and* the family violence literature—is central to the devastating experiences of Bernadette and her daughters during a period of risk and family crisis. Bernadette’s requests for basic information and support, for example, were met with requirements that she engage with Katie’s school via what proved to be impenetrable communication practices overseen by male gatekeepers positioned to placate, patronise or block altogether the voice of a mother attempting to advocate for her daughter’s safety and wellbeing. When asked how she herself accounted for her treatment by male gatekeepers, Bernadette noted:

I think there’s a real element of sexism that exists in how schools deal with family separation…I really felt like I was just dismissed as this hysterical stupid housewife, and their dad was, it was you know ‘oh poor dad, you know he just wants to see his kids, that’s why we’re letting him have private meetings with your daughter on school grounds.’ …it sort of felt like they were doing everything they could to help him, and just ignoring me. And I thought, is this a sexist thing? …I just did not understand why from the get-go I was this hysterical ranting woman, and he was this poor victim who’s been victimised by a feminist court system …And I felt that from the school, and I just sort of thought something’s not right here.

What Bernadette’s experience documents is that these were neither isolated incidents nor interactions with one or two individuals with limited knowledge of family violence issues. Rather, they were attitudes and patterns of engagement encountered across multiple interactions with various staff at two separate schools. Her concerns about child safety and wellbeing were repeatedly trivialised or ignored, as were Katie’s emotional distress and self-harming, while the agenda of their abuser was given priority at multiple points by key stakeholders, including Katie’s school counsellor:

…knowing full well that Katie was being coached by him and abused by him, [the school counsellor] sat Katie down in their first meeting and told Katie ‘Now, if you want to influence the court case, all of this can be subpoenaed. So everything you say here can be recorded.’ And I was just like ‘Are you helping him to coach her? I don’t understand. Like, are you helping him? He’s an abuser, why are you helping him?’

Already the subject of a court issued Protection Order and permitted supervised-only contact with his children at the time of the above incident, Bernadette’s former partner’s attempts at paper abuse and procedural stalking found sympathetic ground in school cultures that persistently prioritised the voice of even violent men over the voice of women. Kenway and Fitzclarence place such cultures decisively within the terms of school complicity in the production of violence:

...if [schools] rationalise violence then they are complicit. If they are structured in such a way as to endorse the culture of male entitlement and indicate that the needs of males are more important than those of females then they are complicit (Kenway and Fitzclarence 2005, p. 46).

In Bernadette’s story, violent and abusive behaviour rationalised as ‘fatherhood wronged’, gives way to violent and abusive behaviour enabled and facilitated by complicit school stakeholders, who occupied positions of leadership and responsibility from which they might otherwise have made a significant difference to a vulnerable child’s safety and wellbeing. Family violence, its ongoing impacts on women and children, and its discursive effacement as a private rather than a social problem thus become embedded in the everyday cultural practices of schools.

Indeed, Bernadette raised the issue of gender several times during her interview, including in relation to others apart from herself and her children. She noted how, for example, Nina’s female teacher at the primary school had been left to fend for herself on the afternoon the children’s father had arrived at school and taken the children:

…where they failed was, they knew there was a Protection Order in place, they knew he wasn’t meant to go to the school, they knew the two younger girls were named on that order as protected persons, and when he marched in and caused a confrontation with [Nina’s] teacher, because she was, obviously knew the circumstances and was confused and caught off guard and he was being quite aggressive and confrontational, um, no one stepped in to assist her, or to support her, or to say, ‘Hang on, this is my school, and this is the order we’ve been given, and no you can’t leave with the children.’ No one did that. He just walked away with them.

Bernadette’s explanation of the gendered nature of these events corresponds with recent research showing that among teachers, there is a perceived lack of support when dealing with violence (McMahon et al. 2017), and that women experience greater levels of parental bullying and harassment than their male counterparts (Billett et al. 2019). Confronted with an aggressive male parent, and in the absence of administrative or collegial support at the time the incident occurred, Nina’s teacher had little option but to attempt, albeit unsuccessfully, to intervene on her own. As some studies have recognised, ‘Since a large proportion of school staff are women, in situations where school staff have to manage a violent parent and protection of a child it is often women who are forced to manage violent men’ (Eriksson et al. 2013a, p. 87).

When asked what she saw as the explanation for both schools’ handling of these events, Bernadette replied that two things stood out in particular—the attitude of family matters being unrelated to school matters, and the issue of gendered ways of seeing and relating to women in circumstances such as hers:

I think first of all there’s an attitude of, ‘Oh that’s not our business, that’s family business. Take that up with the court, we don’t want anything to do with that. That’s too messy for us.’ I also think, based on my experience…dealing with senior school staff, I think there’s a real ‘Oh this woman’s just a raving crazy mother. She’s just one of those mothers who’s putting her ex through hell.’ I honestly felt like that was the attitude that I got…Yeah, it just seemed like they were very dismissive of it. And it was like ‘I’m trying to tell you this man is dangerous, and I’m trying to tell you, I have courts, now two courts and two separate magistrates agree with me by putting these things in force, and no one’s listening.

When understood against the broader context of family violence that in Australia claims the lives of about one woman per week (AIHW 2019), and where children make up 15% of domestic homicides (AIHW 2019), Bernadette’s assessment of her experiences brings into sharp relief what is at stake when families fearful for their safety seek the support and assistance of schools. In the discursive hierarchy of schools attended by Katie, Narelle and Nina, an apparent lack of understanding about family violence issues combined with gendered attitudes, silences and inaction and critical failures of policy, procedure and practice, significantly compromised the safety and wellbeing of a mother and three children.

## Conclusions

The prelude to this paper calls for a national conversation about school policies, procedures and everyday practices when dealing with children and parents living with or fleeing family violence. As educators, researchers and parents, we understand and value the importance that many schools place on student safety, and on their interactions with and support for children, parents and families. We also recognise the challenges associated with balancing the many responsibilities of schools, and the complexities that arise when family violence intersects with the role of schools. What we hope to accomplish in this case study analysis is, firstly, to highlight these complexities and the risks to children and families when educators and school leaders are insufficiently prepared for and poorly supported in responding to these risks. We hope, secondly, to highlight and call to account the kinds of cultural practices, discursive silences and gendered power relations that can render schools complicit in the production and perpetuation of family violence. Finally, we hope to initiate an ongoing, interdisciplinary dialogue that focuses on ways that education researchers and practitioners can rethink taken for granted norms and assumptions about the role of schooling and education research as sites of intervention, safety and support for women and children affected by family violence.

## Postscript

As noted in the methodology section, Bernadette reviewed this manuscript prior to submission to check for accuracy and participant confidentiality. With her permission, we conclude with her remarks:

‘All factually correct. A bit sad to look back on it, but I hope it sparks the conversation that leads to change’.

## Footnotes

1 Pseudonyms are used for all names of individuals, schools and cities/towns.

2 We use the term ‘family violence’ throughout this paper, in line with guidance from the Royal Australian College of General Practitioners (RACGP). The term encompasses a range of other terms such as domestic violence, intimate partner abuse and child abuse, and includes ‘any violence or abuse that is occurring within a family’ (RACGP 2014, p. 2). Family violence may include some, or all, of the following: physical abuse, emotional abuse, child sexual abuse, adult sexual abuse, economic abuse, social abuse or neglect, all of which ‘involve an abuse of power’ (RACGP 2014, p. 3).

3 Human Research Ethics Committee, University of Southern Queensland. H18REA239.

4 Australian Council of State School Organisations, Australian Catholic Schools Parents, Australian Parents’ Council.

5 The term ‘Protection Orders’ will be used here, reflecting the nomenclature in the state in which the research obtained university ethics approval. The use of this term should not be taken as an indication of the location of participants prior to, during or after the events described in this article.

## References

[CR1] Aitchison, C. (2006). *Mothers and school choice: Effects on the home front.* Unpublished doctoral thesis, Faculty of Education: University of Technology, Sydney. Retrieved from https://opus.lib.uts.edu.au/bitstream/10453/20058/1/01front.pdf.

[CR2] Aitchison C, Goodwin S, Huppatz K (2010). Good mothers go school shopping. The good mother: Contemporary motherhoods in Australia.

[CR3] Arnot M (2002). Reproducing gender? Essays on educational theory and feminist politics.

[CR4] Australian Bureau of Statistics. (2016). Report 4906.0 – Personal safety in Australia, 2016. Retrieved from https://www.abs.gov.au/ausstats/abs@.nsf/mf/4906.0.

[CR5] Australian Bureau of Statistics. (2017). Report 3310.0 – Marriages and divorces, Australia, 2016. Retrieved from https://www.abs.gov.au/ausstats/abs@.nsf/7d12b0f6763c78caca257061001cc588/8d7e63106b9b30caca257d9b000ba3f5!OpenDocument.

[CR6] Australian Bureau of Statistics. (2019). 3310.0 Marriages and Divorces, Australia, 2018. Retrieved from https://www.abs.gov.au/statistics/people/people-and-communities/marriages-and-divorces-australia/latest-release#data-download.

[CR8] Australian Bureau of Statistics (2020). Partner violence – in focus: Crime and justicestatistics. 28 January 2020. https://www.abs.gov.au/statistics/people/crime-and-justice/focus-crime-and-justice-statistics/latest-release.

[CR9] Australian Domestic & Family Violence Death Review Network. (2018). Data Report. Australian Domestic & Family Violence Death Review Team. Retrieved from https://www.coroners.justice.nsw.gov.au/Documents/ADFVDRN_Data_Report_2018%20(2).pdf.

[CR10] Australian Institute of Health and Welfare. (2019). Family, Domestic and Sexual Violence in Australia: Continuing the National Story 2019—In brief. Cat. no. FDV 4. Canberra: AIHW. Retrieved from https://www.aihw.gov.au/getmedia/b180312b-27de-4cd9-b43e-16109e52f3d4/aihw-fdv4-FDSV-in-Australia-2019_in-brief.pdf.aspx?inline=true

[CR12] Barr, J. & Saltmarsh, S. (2014). ‘It all comes down to the leadership’: The role of the school principal in fostering parent-school engagement.* Educational Management, Administration and Leadership, 42*(4), 491–505. 10.1177/1741143213502189

[CR17] Beck CJA, Raghavan C (2010). Intimate partner abuse screening in custody mediation: The importance of assessing coercive control. Family Court Review.

[CR18] Billett, P., Fogelgarn, R., & Burns, E. (2019). *Teacher targeted bullying and harassment by students and parents: Report from an Australian exploratory survey*. LaTrobe University. Retrieved from https://www.ttbhau.com/uploads/2/0/8/1/20818406/final_ttbh_report_april_20_2019.pdf .

[CR19] Blackmore J, Hutchison K (2010). Ambivalent relations: The ‘tricky footwork’ of parental involvement in school communities. International Journal of Inclusive Education.

[CR15] Brickell, K., & Garrett, B. (2015). Storytelling domestic violence: Feminist politics of participatory video in Cambodia. *ACME: An International E-Journal for Critical Geographies, 14*(3), 928–953.

[CR20] Butler A, Buxton-Namisnyk E, Beattie S, Bugeja L, Ehrat H, Henderson E, Lamb A, Dawson M (2017). Australia. Domestic homicides and death reviews: An international perspective.

[CR21] Callaghan JEM, Alexander JH, Sixsmith J, Chiara Fellin L (2018). Beyond “witnessing”: Children’s experiences of coercive control in domestic violence and abuse. Journal of Interpersonal Violence.

[CR22] Carlson M, Wittrup E, Moylan C, Ortiz D (2020). A good call? Contextual factors influencing mandated reporting in domestic violence programs. Journal of Family Violence.

[CR23] Cattaneo LB, Cho S, Botuck S (2011). Describing intimate partner stalking over time: An effort to inform victim-centered service provision. Journal of Interpersonal Violence.

[CR24] Certeau, M. (1984). *The practice of everyday life*. (S. F. Rendall, Trans.). University of California Press. Retrieved from https://www.ucpress.edu/book/9780520271456/the-practice-of-everyday-life.

[CR25] Chang, C. (2020, February 21). Calls for changes to publication of statistics around violent deaths of women and children. *news.com.au*. Retrieved from https://www.news.com.au/national/politics/calls-for-changes-to-publication-of-statistics-around-violent-deaths-of-women-and-children/news-story/00db1453145a28d1e6555d92f39947e8.

[CR26] Colpin H, Vandemeulebroecke L, Ghesquière P (2004). Supporting the educational career of children from divorced families: Parents’ experiences and the role of the school. British Journal of Sociology of Education.

[CR27] Cooper D, Perkins K, Couper G (2012). Family law issues and schools: Guidance for Australian educators. International Journal of Law and Education.

[CR28] Cullen D, Fritzon K (2019). A typology of familicide perpetrators in Australia. Psychiatry, Psychology and Law.

[CR29] Davies S, Berger E (2019). Teachers’ experiences of responding to students’ exposure to domestic violence. Australian Journal of Teacher Education.

[CR30] Declercq F, Meganck R, Audenaert K (2016). A case study of paternal filicide-suicide: Personality disorder, motives, and victim choice. The Journal of Psychology.

[CR31] DeKeseredy W, Dragiewicz M, Schwartz M (2017). Abusive endings: Separation and divorce violence against women.

[CR32] Ellis J, Downe S, Farrelly N, Hollinghurst S, Stanley N, Stanley N, Humphreys C (2015). School-based prevention and the disclosure of domestic violence. Domestic violence and protecting children: New thinking and approaches.

[CR33] Eriksson M, Bruno L, Näsman E (2013). Family law proceedings, domestic violence and the impact upon school: A neglected area of research. Children & Society.

[CR34] Eriksson M, Bruno L, Näsman E (2013). Domestic violence, family law and school: Children’s rights to participation, protection and provision.

[CR89] Fine M (2012). Troubling calls for evidence: A critical race, class and gender analysis of whose evidence counts. Feminism & Psychology.

[CR35] Foucault M (1982). The subject and power. Critical Inquiry.

[CR36] Frauenholtz S, Mendenhall A, Moon J (2017). Role of school employees’ mental health knowledge in interdisciplinary collaborations to support the academic success of students experiencing mental health distress. Children & Schools.

[CR37] Gillies V (2006). Working class mothers and school life: Exploring the role of emotional capital. Gender and Education.

[CR38] Goodall J (2019). Parental engagement and deficit discourses: Absolving the system and solving parents. Educational Review.

[CR16] Greenberg, L. R., Gould-Saltman, D. J., & Schnider, R. (2006).The problem with presumptions—A review and commentary. *Journal of Child Custody: Applying Research to Parenting and Assessment Practice and Policies, 3*(3–4), 139–172. 10.1300/j190v03n03_07.

[CR39] Hamby, S. L., Finkelhor, D., Turner, H., & Ormrod, R. (2011).Children's exposure to intimate partner violence and other family violence. U.S. Dept. of Justice, Office of Justice Programs, Office of Juvenile Justice and Delinquency Prevention. doi: 10.1037/e725322011-001.

[CR40] Hutchison K (2012). A labour of love: Mothers, emotional capital and homework. Gender and Education.

[CR41] Jaffe P, Campbell M, Reif K, Fairbairn J, David R, Dawson M (2017). Children killed in the context of domestic violence: International perspectives from death review committees. Domestic homicides and death reviews: An international perspective.

[CR42] Kainz K, Aikens N (2007). Governing the family through education: A genealogy on the home/school relation. Equity & Excellence in Education.

[CR43] Katz E (2016). Beyond the physical incident model: How children living with domestic violence are harmed by and resist regimes of coercive control. Child Abuse Review.

[CR44] Katz E, Nikupeteri A, Laitinen M (2020). When coercive control continues to harm children: Post-separation fathering, stalking and domestic violence. Child Abuse Review.

[CR45] Kelly J, Johnson MP (2008). Differentiation among types of intimate partner violence: Research update and implications for interventions. Family Court Review.

[CR46] Kenway J, Fahey J (2008). Melancholic mothering: mothers, daughters and domestic violence. Gender and Education.

[CR47] Kenway J, Fitzclarence L, Skelton C, Francis B (2005). Masculinity, violence and schooling: Challenging ‘poisonous pedagogies’. A feminist critique of education: 15 years of gender education.

[CR48] La Kam AB (2016). The critical balance between parents’ rights and students’ safety – How parental kidnapping poses an acute threat to school security. Child and Family Law Journal.

[CR49] Lapierre S (2008). Mothering in the context of domestic violence: The pervasiveness of a deficit model of mothering. Child & Family Social Work.

[CR50] Lapierre S, Featherstone B, Hooper CA, Scourfield J, Taylor J (2010). Are abused women “neglectful” mothers? A critical reflection based on women’s experiences. Gender and child welfare in society.

[CR51] Lapierre S, Côte I, Lambert A, Buetti D, Laergne C, Damant D, Couturier V (2018). Difficult but close relationships: Children’s perspectives on relationships with their mothers in the context of domestic violence. Violence Against Women.

[CR52] Leyton D, Rojas MT (2017). Middle-class mothers’ passionate attachment to school choice: Abject objects, cruel optimism and affective exploitation. Gender and Education.

[CR53] Lloyd M (2018). Domestic violence and education: Examining the impact of domestic violence on young children, children, and young people and the potential role of schools. Frontiers in Psychology.

[CR54] Luttrell W (1997). School-smart and mother-wise: Working-class women’s identity and schooling.

[CR55] Macfarlane K (2008). Playing the game: Examining parental engagement in schooling in postmillennial Queensland. Journal of Education Policy.

[CR56] Macfarlane K (2009). Navigating a treacherous game: Conceptualising parental engagement in contemporary Queensland schooling. British Journal of Sociology of Education.

[CR57] McGowan, M., & Smee, B. (2020). Queensland police spark anger with 'open mind' comment on murder of Hannah Clarke and children *The Guardian.* Retrieved from https://www.theguardian.com/australia-news/2020/feb/20/queensland-police-spark-anger-with-open-mind-comment-on-of-hannah-clarke-and-children?fbclid=IwAR0K52WXbT76oeXCM8hT4bndfvy1qvvPmUHlMYJjKH9rM5hOkRB8hVCso6Y.

[CR58] McKay J, Garratt D (2013). Participation as governmentality? The effect of disciplinary technologies at the interface of service users and providers, families and the state. Journal of Education Policy.

[CR59] McMahon S, Reaves S, McConnell E, Peist E, Ruiz L (2017). The ecology of teachers’ experiences with violence and lack of administrative support. American Journal of Community Psychology.

[CR60] Meyer S, Frost A (2019). Domestic and family violence: A critical introduction to knowledge and practice.

[CR61] Miller S, Smolter N (2011). ‘Paper abuse’: When all else fails, batterers use procedural stalking. Violence Against Women.

[CR62] Monk, L. (2017). *Improving professionals' responses to mothers who become, or are at risk of becoming, separated from their children, in contexts of violence and abuse*. Department of Psychology and Behavioural Sciences, Coventry, UK: Coventry University. Retrieved from https://curve.coventry.ac.uk/open/file/1ca9a999-261e-4434-a8d3-c60e38f39248/1/Binder1.pdf .

[CR63] Morgan, A., & Chadwick, H. (2009). *Key issues in domestic violence*. Research in Practice Summary Paper No. 07*.* Australian Institute of Criminology. Retrieved from https://aic.gov.au/publications/rip/rip07.

[CR64] Oates K, Merkel-Holguin L, Fluke J, Krugman R (2018). Child protection systems in Australia. National systems of child protection.

[CR66] O’Brien M (2008). Gendered capital: Emotional capital and mothers’ care work in education. British Journal of Sociology of Education.

[CR67] O’Hagan K (2014). Filicide-suicide: The killing of children in the context of separation, divorce and custody disputes.

[CR65] Organisation for Economic Cooperation and Development. (2015). OECD Family Database, *SF3.2 Family Dissolution and Children*, OECD - Social Policy Division - Directorate of Employment, Labour and Social Affairs. Retrieved October 22, 2019 from https://www.oecd.org/els/family/SF_3_2_Family_dissolution_children.pdf.

[CR68] Osgood J, Robinson KH (2019). Feminists researching gendered childhoods: Generative entanglements.

[CR69] Peters E (2012). I blame the mother: Educating parents and the gendered nature of parenting orders. Gender and Education.

[CR70] Royal Australian College of General Practitioners (2014). Abuse and violence: Working with our patients in general practice.

[CR11] Saltmarsh, S. (2012) The ‘kid most likely’: Naming, brutality and silence within and beyond school settings. In S. Saltmarsh, K. H. Robinson & C. Davies (Eds.), *Re/thinking school violence: Theory, gender, context* (pp. 21–37). Palgrave. 10.1057/9781137015211_2

[CR91] Saltmarsh S (2015). Michel de Certeau, everyday life and policy cultures: The case of parent engagement in education policy. Critical Studies in Education.

[CR92] Saltmarsh S, McPherson A (2019). Un/satisfactory encounters: Communication, conflict and parent-school engagement. Critical Studies in Education.

[CR71] Saunders D, Oglesby K (2016). No way to turn: Traps encountered by many battered women with negative child custody experiences. Journal of Child Custody.

[CR72] Schandorph Løkkegaard S, Hansen NB, Wolf NM, Elklit A (2019). When daddy stalks mommy: Experiences of intimate partner stalking and involvement of social and legal authorities when stalker and victim have children together. Violence Against Women.

[CR73] Schneider FD, Loveland Cook CA, Salas J, Scherrer J, Cleveland IN, Burge SK (2020). Childhood trauma, social networks, and the mental health of adult survivors. Journal of Interpersonal Violence.

[CR74] Shuffleton A (2017). Parental involvement and public schools: Disappearing mothers in labor and politics. Studies in Philosophy and Education.

[CR75] Skinner T, Hester M, Malos E (2013). Researching gender violence: Feminist methodology in action.

[CR76] Stanley, N. (2011). *Children experiencing domestic violence: A research review.* Dartington Research in Practice. Retrieved from https://www.menoresyviolenciadegenero.es/documentos/estudios-sobre-menores-expuestos-a-violencia-de-genero/Children-Experiencing-Domestic-Violence-A-Research-Review.pdf.

[CR77] Stanley N, Miller P, Foster H (2012). Engaging with children’s and parents’ perspectives on domestic violence. Child & Family Social Work.

[CR78] Sterne A, Poole L (2010). Domestic violence and children: A handbook for schools and early years settings.

[CR79] Truu, M. (2020). Queensland police apologise for ‘victim blaming’ comments after murder of Hannah Clarke and kids. *SBS News.* Retrieved from https://www.sbs.com.au/news/queensland-police-apologise-for-victim-blaming-comments-after-murder-of-hannah-clarke-and-kids.

[CR80] Vincent C (2017). ‘The children have only got one education and you have to make sure it’s a good one’: Parenting and parent-school relations in a neoliberal age. Gender and Education.

[CR81] Vincent C, Ball S (2007). ‘Making up’ the middle-class child: Families, activities and class dispositions. Sociology.

[CR82] Vincent C, Ball S, Braun A (2010). Between the estate and the state: Struggling to be a ‘good’ mother. British Journal of Sociology of Education.

[CR83] Vincent C, Martin J (2002). Class, culture and agency: Researching parental voice. Discourse: Studies in the Cultural Politics of Education.

[CR84] Vincent C, Tomlinson S (2006). Home-school relationships: ‘The swarming of disciplinary mechanisms’?. British Educational Research Journal.

[CR85] Webster RS, Whelen J (2019). Rethinking reflection and ethics for teachers.

[CR86] Weis L, Marusza J, Fine M (1998). Out of the cupboard: Kids, domestic violence, and schools. British Journal of Sociology of Education.

[CR87] Woolley, S., & McElroy, N. (2020, February 21). Queensland Police apologises over comments made after Baxter family tragedy. *7news.com.au*. Retrieved from https://7news.com.au/news/qld/police-apology-over-dead-mum-comment-c-708988?fbclid=IwAR1j7gSbD4PdIYFnY9B9AQ567q8qplUAN-Y7o8NbuQe4hJzXChM7JNh0wsE.

[CR88] Youdell D (2019). Judith Butler and education.

